# Structural validity and measurement invariance of the Japanese version of the Kessler Psychological Distress Scale (K6): an item response theory analysis

**DOI:** 10.1080/07853890.2026.2725314

**Published:** 2026-09-03

**Authors:** Chihaya Machida, Hiroyuki Uchida, Takumi Igusa, Tomohiro Shimada, Kazuki Hirao

**Affiliations:** a Department of Rehabilitation, Fujioka General Hospital, Fujioka, Japan; b Department of Rehabilitation, Kurashiki Heisei Hospital, Kurashiki, Japan; c Graduate School of Health Sciences, Gunma University, Maebashi, Japan; d Department of Rehabilitation, Uchida Hospital, Numata, Japan; e Department of Occupational Therapy, School of Health Sciences, Faculty of Medicine, Kagoshima University, Kagoshima, Japan

**Keywords:** Psychological distress, Kessler Psychological Distress Scale, structural validity, item response theory, measurement invariance

## Abstract

**Background:**

The Kessler Psychological Distress Scale (K6) is in widespread use to screen for psychological distress, but evidence to support the quality of the Japanese version (JK6) remains limited, in particular with respect to item response theory (IRT)-based item parameters and the test information function (TIF). Evidence concerning measurement invariance across gender and age groups likewise remains limited. Therefore, this study examined the structural validity and measurement invariance of the JK6 in an online community sample of 6632 Japanese-speaking adults aged 20–59 years.

**Materials and methods:**

Confirmatory factor analysis (CFA) was used to compare one- and two-factor models. Using IRT with a graded response model, item discrimination and threshold parameters were estimated and item information functions and the TIF were derived. Measurement invariance across gender and age groups was tested using multi-group CFA (MCFA), and differential item functioning (DIF) was examined via ordinal logistic regression.

**Results:**

The CFA results supported a primarily unidimensional interpretation of the JK6. IRT analyses showed very high item discrimination, and the TIF indicated high measurement precision for mild-to-moderate levels of psychological distress. MCFA supported scalar invariance across gender and age groups, and the DIF effect sizes were negligible.

**Conclusions:**

These findings indicate that the JK6 is a primarily unidimensional measure of psychological distress that provided precise and comparable assessment across gender and age groups in an online community sample of Japanese-speaking adults aged 20–59 years. Further studies should be undertaken to examine the generalizability of these findings across the broader Japanese adult population, including older adults and clinical populations.

## Key Messages

- Although the Kessler Psychological Distress Scale (K6) is in widespread use to screen for psychological distress, there is only limited evidence for the Japanese version, particularly regarding structural validity, IRT-based item characteristics, measurement precision and measurement invariance.
- In an online community sample of Japanese-speaking adults aged 20–59 years, the Japanese version of the K6 (JK6) presented a primarily one-factor structure, very high item discrimination, high measurement precision at mild-to-moderate levels of psychological distress, and measurement invariance across gender and age groups.
- These findings support the use of the JK6 to assess psychological distress in online community samples of Japanese-speaking adults aged 20–59 years.

## 1. Introduction

Psychological distress is a multifaceted construct including nonspecific symptom domains, such as depression and anxiety [1,2]. Psychological distress is a common clinical feature of many mental disorders and has been shown to have a serious impact on mental well-being even when it is not high enough to be diagnosed as a mental disorder [1,3,4]. It has been associated with a range of physical and mental health problems, including increased mortality, a higher risk of developing cardiovascular disease, cancer and diabetes, a reduced quality of life, diminished subjective well-being, and weakened social support [4–8]. Although psychological distress is a serious health issue for both individuals and society, it has been insufficiently recognized or evaluated in clinical and community settings [9]. There is a need for assessment scales that are simple, reliable, valid and suitable for screening in clinical settings and communities to detect psychological distress at an early stage and connect individuals with appropriate support [4].

Several scales have been developed to assess psychological distress. Among them, the Kessler Psychological Distress Scale (K6) developed by Kessler et al. may be the most appropriate scale for early and accurate assessment of psychological distress [10]. The K6 consists of six items, each rated on a Likert scale of 0–4, with a total score ranging from 0 to 24 points, with higher scores indicating more severe psychological distress. The scale’s simplicity, high reliability and validity have led to its widespread international recognition as an excellent screening tool. Previous studies have verified the characteristics and usefulness of K6 in countries with different cultural and social backgrounds, such as the United States, Australia, China, Vietnam, Canada and Japan [2,3,11–18].

The structural validity of the K6 has also been investigated in many countries. Confirmatory factor analysis (CFA) has been used to propose a one-factor model consisting of psychological distress and a two-factor model consisting of depression and anxiety. The one-factor model is supported by research in the United States, Australia, China and Vietnam [11,12,14,19]. Meanwhile, studies targeting young people in Hong Kong, Canada, and certain clinical groups have reported that a two-factor model that distinguishes between depression and anxiety is more appropriate [15,16,20]. Opinions on the optimal factor structure therefore seem to differ depending on the characteristics and cultural background of the target population.

Although the Consensus-Based Standards for the Selection of Health Measurement Instruments (COSMIN) recommends verifying the validity of each measurement property, no studies appear to have examined the structural validity of the Japanese version of the K6 (JK6) using CFA or to have reported its item characteristics and measurement precision based on item response theory (IRT) [21,22]. For this reason, this study assessed the structural validity of the JK6 in an online community sample of Japanese-speaking adults aged 20–59 years using CFA, and it clarified its item characteristics and measurement precision with the use of IRT.

## 2. Materials and methods

### 2.1. Study design

This study employed a cross-sectional design to examine the structural validity, internal consistency, item characteristics based on IRT, and measurement invariance of the JK6. The data analysed in this study were obtained from prior studies conducted by Uchida et al. involving Japanese adult populations and were collected online [23–25]. Data collection was conducted in December 2020. All participants provided informed consent prior to participation. The study protocol was approved by the Gunma University Ethical Review Board for Medical Research Involving Human Subjects (Approval No. HS2020-168). This study was conducted in accordance with the ethical principles of the Declaration of Helsinki. All evaluation and analysis procedures followed the guidelines of the COSMIN, which provides internationally accepted criteria for the assessment of health-related measurement properties [21,22].

### 2.2. Participants

The study participants were recruited from a panel list maintained by Marketing Applications Inc. (Tokyo, Japan). An email including information on the survey and an option to provide informed consent was delivered to native Japanese-speaking adults aged 20–59 years. Individuals who agreed to participate by clicking the ‘I agree’ button were enrolled in the survey. The questionnaire that was used in this study was configured to automatically reject incomplete responses, so that missing data were automatically eliminated. Thus, the responses from all participants were included in the statistical analysis.

### 2.3. Instrument

The K6 is a six-item self-report scale that measures the severity of psychological distress over the past 30 days [10]. Each item is rated on a five-point scale (4 = all the time, 3 = most of the time, 2 = sometimes, 1 = a little of the time, and 0 = none of the time), and the total score ranges from 0 to 24. Higher scores indicate greater psychological distress. In this study, the JK6 was used for evaluation [17].

### 2.4. Statistical analysis

In addition to classical test theory (CTT), IRT was also used for psychological measurement evaluation [21,22]. These approaches were adopted to evaluate multiple measurement properties of the JK6, including its internal consistency, structural validity, IRT-based item characteristics, precision of measurement, and measurement invariance. All of the statistical analyses were performed using R (version 4.4.3) (Vienna, Austria).

#### 2.4.1. Structural validity

CFA was conducted to evaluate the structural validity of the JK6 [21,22]. CFA is a method for verifying the extent to which a pre-assumed factor structure fits the data [21,22]. CFA was conducted using the weighted least squares mean and variance-adjusted (WLSMV) estimator, which is suitable for ordinal scale items and large samples [22]. All six of the JK6 items were treated as ordered categorical variables, and WLSMV estimation was adopted using the polychoric correlation matrix and thresholds for the ordinal response categories. Thus, in this analysis, no multivariate normality was assumed for the JK6 items as continuous observed variables. For scale identification, the latent factor variance was fixed to 1, and all of the factor loadings were freely estimated. Standardized factor loadings were estimated and reported to evaluate the strength of the association between each item and the latent factor. Because prior studies have yielded inconsistent findings regarding the dimensionality of the JK6, two competing models were compared. The first was a one-factor model representing psychological distress as a single construct, and the second was a two-factor model that distinguishes between depression and anxiety as related but separate dimensions [2,11,12,14–16,19,20,26]. Although both models have received empirical support, the K6 is typically used as a brief screening tool and interpreted based on the total score in both research and clinical settings [25,27–29]. In line with its conceptual design and practical usage, the one-factor model was considered more parsimonious and theoretically appropriate [10]. Therefore, the one-factor model was hypothesized to best represent the underlying structure of the JK6 in this study. Model fit was assessed using several indices, including the comparative fit index (CFI), Tucker–Lewis index (TLI), root mean square error of approximation (RMSEA), standardized root mean square residual (SRMR) and chi-square (*χ*^2^) [21,22]. A model was considered to have good fit when the CFI and TLI values were 0.95 or above and acceptable fit when they were 0.90 or above [21,22,30,31]. The RMSEA value was interpreted as indicating good fit when it was less than 0.06 and acceptable fit when it was less than 0.08 [21,22,31]. Similarly, the SRMR was regarded as indicating acceptable fit when it was below 0.08 [21,22,31]. The CFA results were also used to assess unidimensionality, a key assumption for conducting IRT analysis. CFA was conducted using the *lavaan* package in *R*.

#### 2.4.2. Item response theory analysis

Prior to IRT analysis, several assumptions needed to be met to ensure the validity of the results [32–35]. Three key assumptions – unidimensionality, local independence and monotonicity – were evaluated using the procedures described below [32–35]. These preliminary assumption tests were conducted using the *psych*, *MBESS*, *mokken* and *mirt* packages for *R*.

##### 2.4.2.1. Unidimensionality

To assess unidimensionality, which reflects the extent to which a scale measures a single latent trait, McDonald’s omega (*ω*) coefficients – both *ω* hierarchical and *ω* total – were calculated alongside the CFA results [32–35]. These represent the proportion of variance attributable to the general factor, with higher values indicating stronger unidimensionality [36]. Furthermore, to assess the essential unidimensionality in greater detail, we calculated the ratio of hierarchical *ω* to total *ω* (*ω*H/*ω*total), the percentage of uncontaminated correlations (PUCs) and the explained common variance (ECV) [37,38]. These indices were used as supplementary evidence to determine whether a unidimensional IRT model was appropriate for the JK6.

##### 2.4.2.2. Local independence

Local independence refers to the assumption that, after controlling for the latent trait, an individual’s response to one item is statistically independent of their responses to other items [39–41]. Yen’s *Q*3 statistic – based on residual correlations between item pairs – was used to test this assumption [22,39]. Local independence was generally upheld when *Q*3 values were below 0.37 [22].

##### 2.4.2.3. Monotonicity

The Hi coefficient was calculated for each item to confirm monotonicity, in which the response probability for each item increases monotonically with an increase in the latent trait level [40]. Monotonicity was deemed to exist if the Hi coefficient was 0.30 or above [22].

##### 2.4.2.4. Item response theory model and parameter estimation

IRT analysis was conducted based on the graded response model (GRM) [42–44]. For each item, the researchers estimated the discrimination parameter (*a*), which specified the probability of response to the item at the individual’s latent trait (*θ*) level, and multiple difficulty parameters (*b*), which indicated the difficulty of each category boundary [42,44]. In clinical and research contexts, a discrimination parameter (*a*) value exceeding 1.0 is generally considered to indicate sufficient discriminative ability [45]. Each difficulty parameter (*b*) indicated the threshold that separates adjacent response categories on the latent trait (*θ*) scale [46]. Higher difficulty values implied that selecting a more severe option required a higher level of psychological distress [46]. These estimates helped clarify how item responses varied according to the level of *θ*. Model fit was evaluated using the *M*2 statistic and CFI, TLI and RMSEA [22]. Good fit was defined as CFI and TLI values of 0.90 or higher and RMSEA of less than 0.08 [22]. To complement the statistical indices, an item information function (IIF) was generated to illustrate the precision of measurement for each item across the latent trait continuum [35,43]. These visual tools supported a more nuanced interpretation of item performance. IRT modelling and parameter estimation were conducted using the *mirt* package.

##### 2.4.2.5. Test information function

To evaluate the overall measurement precision of the JK6, the test information function (TIF) and the standard error of measurement at each level of the latent trait (SE(*θ*)) were calculated using the GRM [43]. The TIF was obtained by summing the IIF across all items, representing the total information provided by the scale across the latent trait continuum [35,47–49]. A higher TIF value corresponded to a lower SE(*θ*), indicating greater measurement precision [35,47–49]. Measurement reliability was also estimated from the TIF using the following formula [21,22]:

Reliability=1−(1/Test Information)

This estimate can be interpreted similarly to Cronbach’s alpha (*α*) and McDonald’s *ω*; for reference, a TIF of 5 corresponded to a reliability of approximately 0.80 [50]. TIF and SE(*θ*) were computed using the *mirt* package.

### 2.5. Internal consistency

Cronbach’s *α* coefficient and McDonald’s *ω* coefficient (*ω* total, *ω* hierarchical) were calculated to evaluate internal consistency [21,22,36]. Acceptable internal consistency was achieved when Cronbach’s *α* and *ω* reached total values of 0.70 or higher, and *ω* hierarchical, which reflected the proportion of common variance explained by a general factor, of 0.50 or higher [21,22,51,52]. To evaluate the precision of the estimates, 95% confidence intervals (CIs) were also calculated for each reliability coefficient. Internal consistency metrics were calculated using the *psych* and *MBESS* packages.

### 2.6. Measurement invariance

#### 2.6.1. Multi-group confirmatory factor analysis

To evaluate the measurement invariance, multi-group confirmatory factor analysis (MCFA) was conducted using gender and age groups by decade (20–29, 30–39, 40–49, 50–59) as grouping variables. MCFA was conducted adopting the same estimation approach as that of the single-group CFA. All six items of the JK6 were assessed as ordinal categorical variables, and the analysis was performed using WLSMV based on a polychoric correlation matrix. While the chi-square difference test (Δ*χ*^2^) was one of the standard approaches for evaluating measurement invariance, it is highly sensitive to sample size and could indicate statistically significant differences even when differences in model fit or parameter estimates are trivial [53]. Therefore, due to the large sample size of this study, we did not employ Δ*χ*^2^ to evaluate measurement invariance. Instead, measurement invariance was evaluated with the use of changes in approximate fit indices, including ΔCFI, ΔRMSEA and ΔSRMR [54]. Invariance was considered supported for successive models when the decrease in CFI was no greater than 0.01 and the increases in RMSEA and SRMR were no greater than 0.015 [54]. MCFA was conducted using the *lavaan* package in R.

#### 2.6.2. Differential item functioning

To evaluate whether item functioning varied by participant attributes (i.e. to examine the fairness of the measurement process), differential item functioning (DIF) was assessed using ordinal logistic regression [55,56]. Three models were compared, including latent trait scores and group variables (gender, age group) and their interactions as predictors. Model 1 included only the latent trait, model 2 added the main effect of the group, and model 3 included the interaction between the latent trait and the group variable. Comparing model fit indices helped clarify the presence of statistically significant overall DIF as well as uniform and non-uniform DIF. Alongside statistical significance, the magnitude of DIF was evaluated by calculating changes in McFadden’s *R*^2^ [22,55]. A change of less than 0.02 was interpreted as negligible [22,55]. These DIF analyses were conducted using the *lordif* package for *R*.

## 3. Results

### 3.1. Characteristics of study participants

Of the 135,848 individuals invited to participate in the survey, 6632 responded, yielding a response rate of 4.9%. Of the 6632 participants included in the analysis, 3321 (50.1%) were male and 3311 (49.9%) were female. The mean age (standard deviation [SD]) was 39.6 (SD = 11.42). Participants were evenly distributed across age groups: 1652 (24.9%) were aged 20–29, 1658 (25.0%) were 30–39, 1656 (25.0%) were 40–49, and 1666 (25.1%) were 50–59 (Table 1).

**Table 1.** Demographic and lifestyle characteristics of the participants.

Variables	*n*	%
Gender		
Men	3321	50.10%
Women	3311	49.90%
Age (decade)	39.6 (11.42)	
20–29	1652	24.91%
30–39	1658	25.00%
40–49	1656	24.97%
50–59	1666	25.12%
Marital status		
Unmarried	3347	50.47%
Married	3285	49.53%
Children		
Without children	3947	59.51%
With children	2685	40.49%
Smoking status		
Non-smoker	5011	75.56%
Current smoker	1621	24.44%
Alcohol use		
Non-drinker	3662	55.20%
Current drinker	2970	44.80%
Regular exercise		
No	4154	62.62%
Yes	2478	37.38%
Educational attainment		
Less than university	3971	59.88%
University degree or higher	2661	40.12%

### 3.2. Descriptive statistics for the JK6

The participants’ mean total score on the JK6 was 5.66 points (SD = 5.79). For all six items, the selection rate for ‘none of the time’ (0 points) was the highest, followed by ‘a little of the time’ (1 point; Table S1).

### 3.3. CFA

To verify the factor structure of the JK6, the fits of one- and two-factor models were compared. The fit indices for the one-factor model were *χ*^2^ (9) = 878.35 (*p* < 0.01), CFI = 0.979, TLI = 0.965 and SRMR = 0.018. While CFI, TLI and SRMR indicated favourable values, RMSEA was 0.127, which did not meet the acceptable criterion (<0.08). Meanwhile, the fit indices for the two-factor model were *χ*^2^ (8) = 828.56 (*p* < 0.01), CFI = 0.980, TLI = 0.962 and SRMR = 0.017. Although the CFI, TLI and SRMR values were favourable even in the two-factor model, the RMSEA was 0.133, which did not meet the acceptance criterion (Table 2). In addition, the estimated latent factor correlation for the two-factor model exceeded 1.0 (*r* = 1.012), which suggests an inadmissible solution and statistical nondistinguishability. From these results, although the CFI, TLI and SRMR were favourable for the two-factor model, the RMSEA did not meet the acceptance criterion, and the estimated latent factor correlation exceeded 1.0. This indicates that the two-factor model could have issues with discriminant validity as the result of an inadmissible solution and statistical non-distinguishability. While the RMSEA value exceeded the criterion for acceptability, it has been shown to be affected by model degrees of freedom and may be inflated in models with few degrees of freedom [57]. Thus, although the elevated RMSEA values for both models warrant caution, the one-factor model was considered more appropriate and parsimonious than the two-factor model for the JK6. The standardized factor loadings for this model ranged from 0.842 to 0.933 (Table 3).

**Table 2.** Confirmatory factor analysis results for the JK6.

Model	*χ* ^2^	df	*p* Value	CFI	TLI	RMSEA	SRMR
One-factor	878.35	9	<0.01	0.979	0.965	0.127	0.018
Two-factor	828.56	8	<0.01	0.980	0.962	0.133	0.017

*χ*^2^: chi-square; df: degrees of freedom; CFI: comparative fit index; TLI: Tucker–Lewis index; RMSEA: root mean square error of approximation; SRMR: standardized root mean square residual.

**Table 3.** Standardized factor loadings for the JK6 derived from CFA.

	One-factor model	Two-factor model	
Item	Psychological distress	Depression	Anxiety
1	0.842		0.841
2	0.933	0.924	
3	0.873	0.867	
4	0.933		0.932
5	0.913		0.912
6	0.906		0.905

### 3.4. IRT metrics

#### 3.4.1. Assumption check and model validity

Before IRT was applied, we tested its key assumptions. First, we adopted CFA to evaluate the fit of a one-factor model and to assess unidimensionality. As noted earlier, although the CFI, TLI and SRMR met acceptable standards, the RMSEA exceeded generally accepted thresholds. Furthermore, the ratio of McDonald’s hierarchical omega to total omega was 0.89, which indicated that the general factor accounted for a substantial portion of the common variance (Table 4). The PUC, which was calculated using the depression and anxiety item classifications that were specified in the two-factor CFA model, was 0.53. In contrast, the ECV for the general factor was 0.91. While the RMSEA and PUC suggested that caution was required for interpreting unidimensionality, previous studies have indicated that ECV and omega hierarchical should also be considered in the evaluation of essential unidimensionality, particularly when PUC is not high [37,38]. Thus, the high *ω*H/*ω*total ratio, the high standardized factor loadings in the one-factor model, and the high ECV supported unidimensionality. The overall pattern of these findings indicated that the unidimensionality assumption of the JK6 was sufficiently supported. Next, the Hi coefficient was calculated for each item to verify the assumption of monotonicity. The results showed no violations of monotonicity, with Hi values ranging from 0.70 to 0.80, indicating that the assumption was met (Table S2). Yen’s *Q*3 statistic was then computed to assess local independence. The *Q*3 values ranged from −0.354 to 0.085, and the absolute values of all item pairs were below the recommended threshold of 0.37, suggesting that the assumption of local independence was upheld (Table S3). These findings supported the appropriateness of applying the IRT model to the JK6.

**Table 4.** Internal consistency of the JK6.

Cronbach’s *α*	95% CI	*ω* total	95% CI	*ω* hierarchical	95% CI
0.94	0.94–0.94	0.94	0.94–0.95	0.84	0.81–0.87

*α*: alpha; CI: confidence interval; *ω*: omega.

#### 3.4.2. Discrimination and difficulty parameters

The estimated discrimination parameters (*a*) and category boundary difficulty parameters (*b*) for each item are summarized in Table 5. The discrimination parameters ranged from 2.913 (item 1) to 5.043 (item 2), indicating that all items demonstrated very high discriminative power. This implies that each K6 item is highly sensitive in differentiating individuals across varying levels of psychological distress. For each item, the difficulty parameters (*b*) showed monotonic increases in the order *b*_1_ < *b*_2_ < *b*_3_ < *b*_4_, representing ordered thresholds along the latent trait (*θ*) continuum.

**Table 5.** Item parameters for the JK6 estimated using the GRM.

Item	*a*	*b*1	*b*2	*b*3	*b*4
1	2.913	–0.066	0.646	1.58	2.199
2	5.043	0.077	0.682	1.413	1.935
3	3.475	0.018	0.728	1.59	2.282
4	5.031	–0.225	0.548	1.273	1.804
5	4.306	–0.112	0.594	1.339	1.893
6	4.187	0.007	0.61	1.256	1.676

*a*: discrimination; *b*: thresholds.

### 3.5. Item and test information

**Figure 1.**
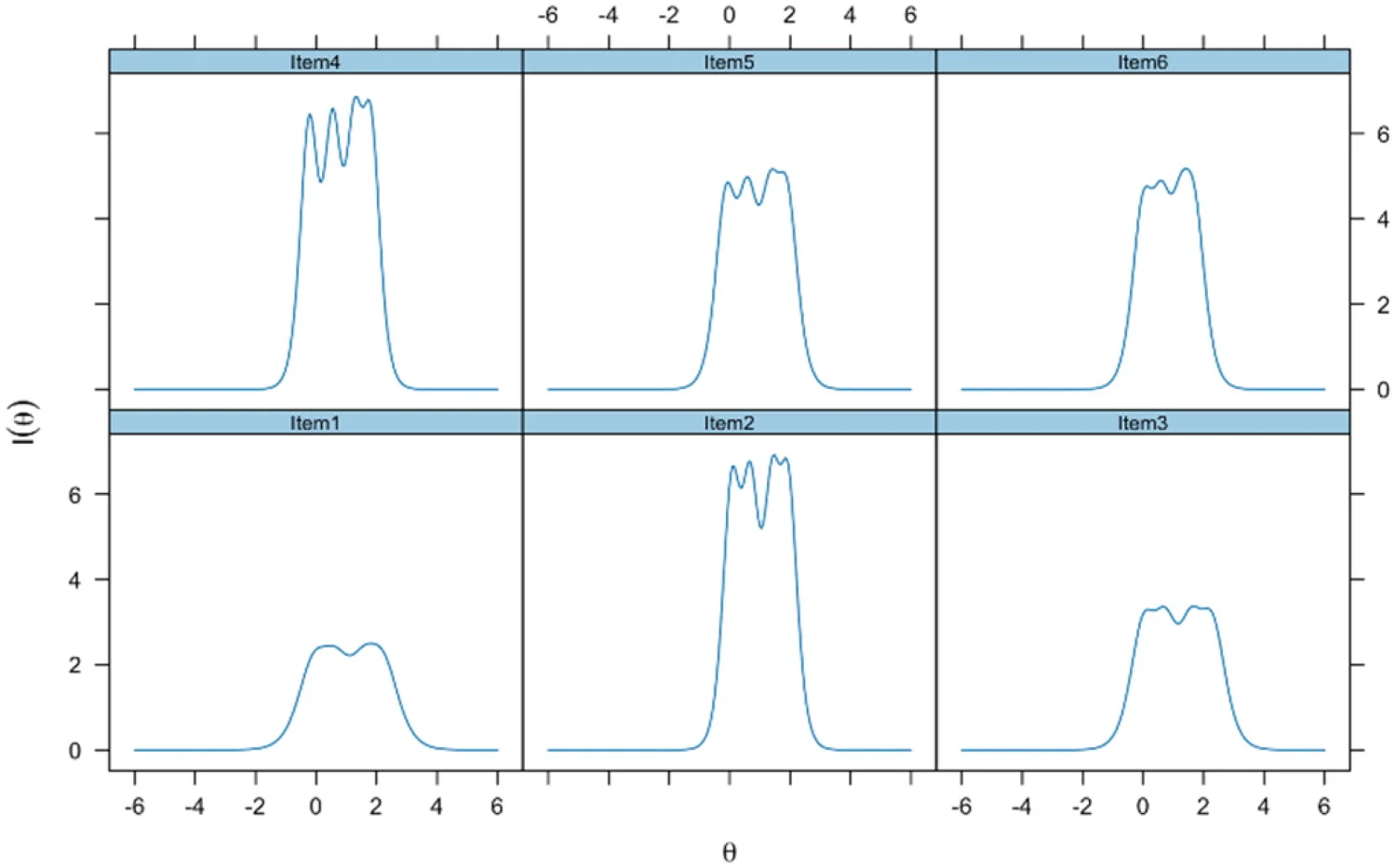
Item information functions for the JK6.

**Figure 2.**
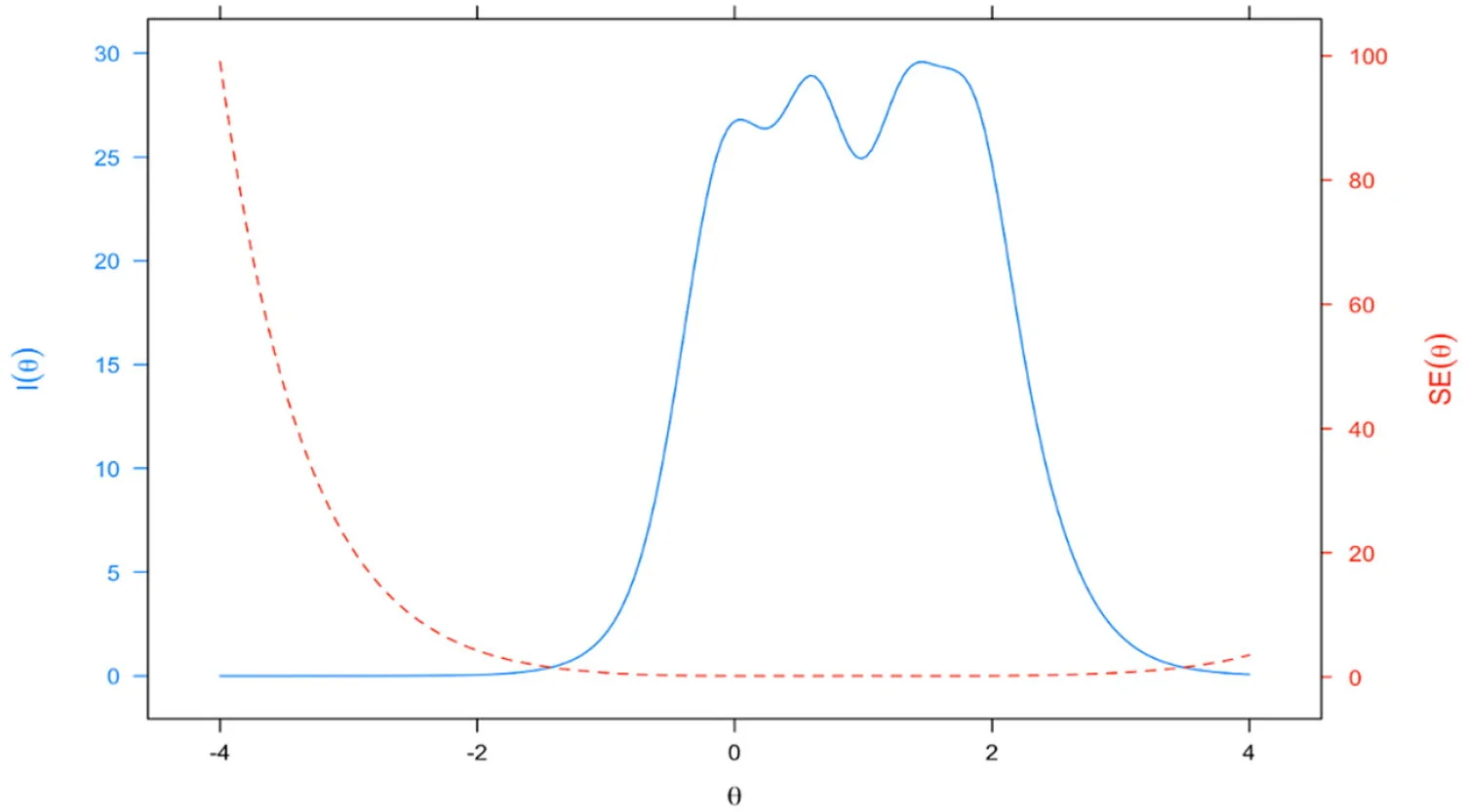
Test information function for the JK6.

The IIF for each item is presented in Figure 1. Many items demonstrated relatively high levels of information within the latent trait (*θ*) range of 0–2. Notably, items 2 and 4 provided greater information than other items in this range. The TIF and standard error of measurement (SE(*θ*)), which reflected the overall measurement precision of the scale, are shown in Figure 2. Using the transformation reliability = 1 − 1/TIF, the scale achieved TIF ≥ 5 from approximately *θ* = −0.76 to 2.68, with a maximum at *θ* = 1.45 (peak reliability = approximately 0.97). These results indicate that the JK6 offers particularly high measurement precision at mildly to moderately elevated levels of psychological distress.

### 3.6. Internal consistency and reliability

Cronbach’s *α* was 0.94 (95% CI: 0.94–0.94), indicating a very high level of internal consistency (Table 4). McDonald’s *ω* total showed a similarly high value of 0.94 (95% CI: 0.94–0.95). In addition, McDonald’s *ω* hierarchical, which reflects the proportion of common variance explained by the general factor, was 0.84 (95% CI: 0.81–0.87), indicating a strong contribution of a single latent construct.

### 3.7. Measurement invariance

Measurement invariance of the one-factor structure of the JK6 was examined across gender (male, female) and age groups (20–29, 30–39, 40–49, 50–59). This entailed the sequential testing of configural, metric and scalar invariance (Table S4). The configural invariance model for the gender group presented favourable values for CFI (0.995), TLI (0.992) and SRMR (0.019); however, the RMSEA was 0.122, and this did not meet the pre-set acceptance criteria, although RMSEA values can be inflated in models that have few degrees of freedom [57]. While the RMSEA value warrants caution, with reference to the favourable CFI, TLI and SRMR values, configural invariance was cautiously supported. Subsequently, models were compared as constraints increased from configural to metric and from metric to scalar and changes were evaluated in CFI, RMSEA and SRMR. All changes satisfied the recommended criteria, with a decrease in CFI no greater than 0.01 and increases in RMSEA and SRMR no greater than 0.015. These results indicated that scalar invariance across gender groups was supported. A similar pattern was observed for age groups. The configural model demonstrated good fit, with CFI at 1.000, TLI at 0.999, SRMR at 0.019, and RMSEA at 0.060. All changes in fit indices between the more and less constrained models met the recommended cutoffs, supporting scalar invariance across age groups.

### 3.8. Examination of DIF

To further examine measurement invariance at the item level, DIF analyses were conducted using ordinal logistic regression across gender and age groups. The results indicated that several items exhibited statistically significant DIF with *p* values of less than 0.01 in both gender and age comparisons. However, the magnitude of DIF, assessed by the change in McFadden’s *R*^2^, was below 0.02 for all items and comparisons (Table 6). This suggests that although some DIF results were statistically significant, their substantive impact was negligible. The JK6 items were therefore considered to function equivalently across gender and age groups.

**Table 6.** DIF analysis results across gender and age groups in the JK6.

Item	Overall DIF (*p*)	ΔMcFadden’s *R*^2^	Uniform DIF (*p*)	ΔMcFadden’s *R*^2^	Non-uniform DIF (*p*)	ΔMcFadden’s *R*^2^
Gender						
1	0.3634	0.0000	0.0001	0.0011	0.0000	0.0004
2	0.0000	0.0023	0.0000	0.0026	0.0246	0.0003
3	0.0000	0.0025	0.0000	0.0028	0.0283	0.0003
4	0.1749	0.0001	0.3978	0.0001	0.9585	0.0000
5	0.0024	0.0005	0.0002	0.0010	0.0050	0.0004
6	0.2329	0.0001	0.2962	0.0001	0.3149	0.0001
Age						
1	0.0118	0.0006	0.0051	0.0011	0.0574	0.0004
2	0.0000	0.0046	0.0000	0.0057	0.0005	0.0011
3	0.0000	0.0015	0.0002	0.0016	0.7045	0.0001
4	0.1202	0.0003	0.1293	0.0005	0.2548	0.0002
5	0.0057	0.0007	0.0095	0.0010	0.2228	0.0002
6	0.0175	0.0006	0.1043	0.0006	0.9414	0.0000

DIF: differential item functioning; Δ: change from previous model.

## 4. Discussion

The results of the CFA indicated that the one-factor model included favourable values for CFI, TLI and SRMR (CFI = 0.979, TLI = 0.965, SRMR = 0.018), although the RMSEA value exceeded the predefined criterion. In addition, the hierarchical *ω* coefficient was 0.84, which indicates that the general factor explained a substantial proportion of the common variance [37,58]. Taken together, these findings suggest that the JK6 functions primarily as a unidimensional scale to measure a single construct, that of psychological distress. This aligned with the theoretical foundation on which K6 was originally developed and its purpose of measuring a general dimension of psychological distress. It also aligned with previous research findings across various countries and populations, including China, Vietnam and Australia [11,12,14]. While the two-factor model also provided favourable values for CFI, TLI and SRMR, the RMSEA value exceeded the predefined criterion, and the inter-factor correlation was excessively high (*r* = 1.012), indicating statistical non-distinguishability. This contradicted findings from several studies conducted in Hong Kong, Canada and Australia [2,16,20]. One possible explanation for this discrepancy lies in differences in the characteristics of the target populations. Previous studies have focused on younger populations (12–25 years old) or populations with clinical disorders [2,16]. In contrast, this study’s sample was formed from an online community sample of Japanese-speaking adults aged 20–59 years. These differences may have prevented the JK6 from being effectively divided into two factors in this study. Future studies should therefore investigate the structural validity of the JK6 in younger populations or those with clinical disorders.

The IRT analysis confirmed that the key assumptions required for applying the IRT model were satisfied. Unidimensionality was supported by favourable fit indices in the CFA (CFI, TLI and SRMR), as well as by a high ratio of McDonald’s *ω* hierarchical to *ω* total. The assumptions of monotonicity and local independence were also met, as indicated by appropriate Hi coefficients and acceptable values of Yen’s *Q*3 statistic, respectively. These results supported the suitability of applying the GRM, which is appropriate for Likert scales, to those in the JK6.

The discrimination parameters (*a*) estimated by the GRM for each item ranged from 2.913 (item 1) to 5.043 (item 2), indicating that all items had a very high ability to differentiate individuals with varying levels of psychological distress. Additionally, the difficulty parameters (*b*_1–4_), which indicated the category boundaries, increased sequentially in the order of *b*_1_ < *b*_2_ < *b*_3_ < *b*_4_ as the level of the latent trait (*θ*) increased. This confirmed that the ordering of the response categories corresponded appropriately to increasing levels of psychological distress.

Analyses of IIF and TIF provided further important insights into measurement precision. TIF values were ≥5 from approximately *θ* = −0.76 to 2.68, indicating that the JK6 measures mild-to-moderate levels of psychological distress with high precision. The TIF peaked around *θ* = 1.45 (peak reliability 0.97). These findings were consistent with the K6’s design objective of increasing reliability as distress increases [10].

The findings of this study can be compared with previous studies that analysed other versions of K6 using IRT. For example, a study of the Vietnamese version of K6 also suggested that item 1 had significantly lower information content [14]. In this study, item 1 of the JK6 also had lower information content than other items, yielding results like those of previous studies [14]. This may suggest that Item 1 of the K6 has low information content regardless of language. Nevertheless, including item 1 may be important for comprehensively evaluating individuals with mild-to-moderate psychological distress and improving the acceptability of responses [14].

MCFA supported scalar invariance across gender and age groups. At the item level, McFadden’s *R*^2^ was less than 0.02 for all items, indicating that measurement bias was essentially negligible. These results support the measurement invariance of the JK6 across gender and age groups, suggesting that item-level measurement bias was negligible in this online community sample of Japanese-speaking adults aged 20–59 years. Comparisons with previous studies showed that measurement invariance was reported in studies targeting adolescents (12–25 years old) and adults in Canada, consistent with the trends observed in this study [15]. However, measurement invariance was not supported in a study targeting 16–25-year-olds in Australia, suggesting that DIF may be more likely to occur during adolescence and young adulthood [2,11]. This may be attributed to the fact that the K6 was originally designed for adults [10]. While measurement invariance is more likely to hold in adult populations, differences in experiences of psychological distress and expression during adolescence and young adulthood may cause the items to function differently across age groups. Cross-cultural differences may also influence item responses. Therefore, future research should use multinational and multicultural designs that are not limited to Japanese samples.

This study had several limitations. First, the survey population was limited to adults aged 20–59 who were registered in an online survey panel; the response rate was relatively low at 4.9% (6632 out of 135,848), so it is not possible to rule out selection bias due to nonresponse. Therefore, caution is required when generalizing the results. Second, this study was based on a nonclinical online community sample rather than on patients who had confirmed diagnoses of mental illness; further examination is therefore required to assess structural validity and measurement precision in clinical populations. Third, the study was limited to individuals aged 20–59, so caution is necessary when generalizing the results to adolescents or older adults. Finally, this study was primarily designed to examine construct validity, and it did not evaluate other measurement characteristics, such as convergent or criterion-related validity. Future research should examine the untested aspects of validity, including convergent and criterion-related validity, including the use of related measures such as the K10 and external criteria such as structured diagnostic interviews and clinical diagnoses.

## 5. Conclusions

This study examined the structural validity of the JK6 using IRT and CTT in an online community sample of Japanese-speaking adults aged 20–59 years. It provides psychometric evidence using a large sample (*n* = 6632), integrating CFA, IRT-based item parameter estimation, test information analysis, and the evaluation of measurement invariance and DIF. The results supported a primarily unidimensional factor structure of the JK6 and demonstrated a high degree of measurement precision across mild-to-moderate levels of psychological distress. Measurement invariance across gender and age groups was supported, indicating that the JK6 assessed psychological distress comparably across these demographic groups in this sample. Overall, these findings indicate that the JK6 has acceptable structural validity, high measurement precision and measurement invariance for assessing psychological distress among Japanese-speaking adults aged 20–59 years recruited from an online community sample. Future research should extend these findings by examining other aspects of validity, including convergent, discriminant, criterion-related and diagnostic accuracy, and by evaluating the scale in adolescents, older adults, clinical populations and samples recruited outside online survey panels.

## Supplementary Material

Supplementary material.docx

## Data Availability

De-identified data are not publicly available due to ethical and privacy restrictions. Aggregate results are reported in the manuscript. De-identified data and analysis code may be made available by the corresponding author upon reasonable request, subject to institutional and IRB approval.
