# Supplementary material for: Structural validity and measurement invariance of the Japanese version of the Kessler Psychological Distress Scale (K6): an item response theory analysis
Source: Ann Med. 2026 Sep 3;58(1):2725314. doi: 10.1080/07853890.2026.2725314 (PMC13543839; doi:10.1080/07853890.2026.2725314)
Supplement: Supplementary file 1 [file IANN_A_2725314_SM9638.docx]

Table S1. Response distributions by JK6 item

| Item | 0 | 1 | 2 | 3 | 4 |
| --- | --- | --- | --- | --- | --- |
| 1 | 3127 (47.15) | 1576 (23.76) | 1405 (21.19) | 343 (5.17) | 181 (2.73) |
| 2 | 3426 (51.66) | 1436 (21.65) | 1226 (18.49) | 346 (5.22) | 198 (2.99) |
| 3 | 3314 (49.97) | 1612 (24.31) | 1259 (18.98) | 320 (4.83) | 127 (1.91) |
| 4 | 2702 (40.74) | 1850 (27.90) | 1360 (20.51) | 460 (6.94) | 260 (3.92) |
| 5 | 2978 (44.90) | 1654 (24.94) | 1322 (19.93) | 442 (6.66) | 236 (3.56) |
| 6 | 3258 (49.13) | 1376 (20.75) | 1186 (17.88) | 442 (6.66) | 370 (5.58) |

Note. 0 = None of the time; 1 = A little of the time; 2 = Sometimes; 3 = Most of the time; 4 = All the time.

Table S2. Monotonicity of the JK6

| Item | 1 | 2 | 3 | 4 | 5 | 6 |
| --- | --- | --- | --- | --- | --- | --- |
| Hi | 0.70 | 0.79 | 0.74 | 0.80 | 0.77 | 0.75 |

Table S3. Yen’s Q3 residual correlation matrix for the JK6

|  | 1 | 2 | 3 | 4 | 5 | 6 |
| --- | --- | --- | --- | --- | --- | --- |
| 1 | 1.0000 | –0.1000 | 0.0850 | –0.2420 | –0.1390 | –0.2500 |
| 2 | –0.1000 | 1.0000 | –0.2270 | –0.2800 | –0.3540 | –0.0640 |
| 3 | 0.0850 | –0.2270 | 1.0000 | –0.2210 | –0.1610 | –0.2320 |
| 4 | –0.2420 | –0.2800 | –0.2210 | 1.0000 | –0.1350 | –0.2350 |
| 5 | –0.1390 | –0.3540 | –0.1610 | –0.1350 | 1.0000 | –0.1370 |
| 6 | –0.2500 | –0.0640 | –0.2320 | –0.2350 | –0.1370 | 1.0000 |

Table S4. Measurement invariance across gender and age groups in the JK6

| Model | CFI | ΔCFI | RMSEA | ΔRMSEA | SRMR | ΔSRMR |
| --- | --- | --- | --- | --- | --- | --- |
| By Gender |  |  |  |  |  |  |
| Configural | 0.995 |  | 0.122 |  | 0.019 |  |
| Metric | 0.995 | 0.000 | 0.122 | 0.000 | 0.020 | 0.001 |
| Scalar | 0.999 | 0.004 | 0.041 | –0.081 | 0.019 | –0.001 |
| By Age |  |  |  |  |  |  |
| Configural | 1.000 |  | 0.060 |  | 0.019 |  |
| Metric | 0.999 | –0.001 | 0.052 | –0.008 | 0.020 | 0.001 |
| Scalar | 0.999 | 0.000 | 0.041 | –0.011 | 0.019 | –0.001 |

Note. CFI: Comparative Fit Index; RMSEA: Root mean square error of approximation; SRMR: standardized root mean square residual; Δ: change from previous model
